# Role of the asthma predictive index (API) in assessing the development of asthma among Brazilian children

**DOI:** 10.1186/1939-4551-8-S1-A61

**Published:** 2015-04-08

**Authors:** Luciana Albuquerque, Virginia PL Ferriani, Ataide Camara, L Karla Arruda, Jorgete Maria Silva

**Affiliations:** 1 Ribeirão Preto Medical School, University of São Paulo, Brazil

## Background

The purpose of the present study was to evaluate the development of asthma in 8-12 year-old children who were seen for an acute wheezing episode in infancy, and to determine the effectiveness of the API in predicting the development of asthma in this group of children.

## Methods

Sixty one of 76 children who participated in a previous study (Camara et al JACI 2004;113;551-7) aimed at identifying risk factors for acute wheezing in infancy were re-evaluated at the ages of 2-4 and 8-12 years. They had been seen at the Emergency Room (ER) for an episode of wheezing at the age of 6-24 months. At the age of 8-12 years, parents completed a questionnaire on respiratory symptoms; 52 children underwent skin prick testing with a panel of inhalant and food allergens and 48 performed methacholine challenge tests. Children were considered asthmatic at the age of 8-12 years if they presented previous physician-diagnosed asthma, or at least one of the following symptoms in the past 12 months: wheezing, cough or chest tightness with exercise, or dry cough without colds, accompanied by bronchial hyperresponsiveness, defined by a PC20 <4mg/ml methacholine challenge test. A positive API (at least one major criteria: physician-diagnosed eczema or parental asthma; or 2 of 3 minor criteria: physician-diagnosed allergic rhinitis, wheezing without colds or peripheral eosinophilia ≥4%) was established based on information collected when the children were 2-4 years-old. Sensitivity, specificity, predictive values, likelihood ratios and confidence intervals (CI) of the API for the diagnosis of asthma at 8-12 years-old were calculated.

## Results

Among the 48 children evaluated at school age, 20 (41.7%) were diagnosed with asthma; 13 of them (65%) had a positive API at 2-4 years. Of the 28 children who did not develop asthma, only 9 (32.1%) had a positive API. Sensitivity and specificity of the API were 65% (CI=40.8-84.6) and 67.9% (CI=47.7-84.1), respectively.

Positive and negative predictive values were 59.1(CI=38.7-79.7) and 73.1 (IC=53.9-86.3); and positive and negative likelihood ratios were 2.02 (CI=1.5-2.73) and 0.51 (CI=0.37-0.72), respectively.

## Conclusions

Asthma at school age was diagnosed in 41.7% of children seen in the ER for acute wheezing in infancy. The chance of developing asthma at school age was two times higher in children with a positive API, as compared with API negative children.

